# Primary Immunodeficiency Registry System: The Minimum Data Set Designing Phase—A Systematic Review and Quantitative Delphi Study

**DOI:** 10.1002/hsr2.71015

**Published:** 2025-07-09

**Authors:** Saman Mohammadpour, Hassan Emami, Sima Shokri, Rafat Bagherzadeh, Soqrat Omari Shekaftik, Zanko Hosseini

**Affiliations:** ^1^ Department of Health Information Technology and Management, School of Allied Medical Sciences Shahid Beheshti University of Medical Sciences Tehran Iran; ^2^ Department of Allergy and Clinical Immunology, Rasool e Akram Hospital Iran University of Medical Sciences Tehran Iran; ^3^ Department of English Language, School of Health Management and Information Sciences Iran University of Medical Sciences Tehran Iran; ^4^ Department of Occupational Health Engineering, School of Public Health Tehran University of Medical Sciences Tehran Iran; ^5^ Students' Scientific Research Center Tehran University of Medical Sciences Tehran Iran; ^6^ Department of Health Information Management, School of Medicine Ardabil University of Medical Sciences Ardabil Iran

**Keywords:** MDS, minimum data set, PID, primary immunodeficiency, registry

## Abstract

**Background and Aims:**

Registries are powerful tools for data management. Designing a minimum data set as the first step in registry development helps collect relevant and efficient data. The aim of this study was to develop a minimum data set for the primary immunodeficiency registry system.

**Methods:**

This cross‐sectional study was conducted at two stages in 2023. In the first stage, primary data elements were extracted from related literature. In the second stage, based on the data elements extracted from the first stage, a questionnaire was developed. Then, using the questionnaire and the Quantitative Delphi Method, the minimal data set on primary immunodeficiency was obtained from 10 asthma and allergy specialists.

**Results:**

In the first stage, the initial minimum data set consisted of 198 data elements, which were categorized into two categories: administrative and clinical. Administrative data were classified into two categories: demographic and patient index. Clinical data were categorized into four categories: patient history, physical examination, tests, and diagnosis. In the second stage, eight elements were removed during the first round of Delphi. One element was removed in the second round of Delphi. In the first round of Delphi, 13 elements were recommended. In the second round of Delphi, all the recommended elements were included in the final list of the minimum data set. Finally, 202 data elements were selected as the final minimum data set.

**Conclusion:**

The created primary immunodeficiency minimum data set is expected to improve decision‐making by clinicians and policymakers, and also improve scientific research in this field.

## 1 Introduction

Primary immunodeficiency (PID) is caused by a genetic defect of the immune system and is a lifelong disease in most cases [1, 2]. Infectious and noninfectious complications are observed among patients with PID. The Infectious complications include frequent and hard‐to‐treat infections, which are not common in healthy individuals. The noninfectious complications include neutropenia, autoimmunity, lymphadenopathy, enteropathy, dermatitis, malignancy, and atopy [3, 4]. There are 555 known primary immune disorders arising from 500 known molecular etiologies [5]. They are classified into 10 groups based on pathogenesis and/or clinical features [6].

Approximately more than one percent of the world population suffers from primary immunodeficiency disease [7], which is more prevalent in the Middle East and North Africa due to the high rate of consanguineous marriages. The rate of consanguineous marriages in the world ranges from 1% to 9%, while in the countries of the Middle East and North Africa, it is reported between 20% and 56%. Consequently, the autosomal recessive mode of inheritance is aggravated, causing an increase in genetic diseases [8]. If primary immunodeficiency is not diagnosed and treated promptly, it may lead to more severe secondary complications, such as life‐threatening infections, developmental disorders, an increased risk of cancer, heart, kidney, and nervous disorders, and ultimately death. This not only causes more deaths, but also incurs higher costs [9].

To ensure timely identification and treatment of this disease, physicians require a wide range of data (including family, social, vaccination, surgery, drug reaction, sensory motor, genetic, clinical, and para‐clinical data) [10]. Therefore, the primary reason for the delayed diagnosis and inadequate treatment of this disease stems from physicians' lack of comprehensive knowledge [5]. Moreover, access to data related to this disease leads to the improvement of epidemiological information and enhances physician training. Furthermore, in light of advancements in data science, these data play a crucial role in research [11]. In countries with weak data collection systems, such as Iran, there is no comprehensive health data recording system. Data are usually collected in an incomplete and fragmented manner in various formats in different organizations. Consequently, there is often no access to or aggregation of data for educational, therapeutic, and research purposes [7]. One solution to address the issue is to establish a disease registry system that accurately and thoroughly collects, stores, and publishes the important and necessary information from different groups [7]. Registries are one of the main tools for disease and hospital data management (including collection, data processing, and information dissemination) that are developed based on the existing clinical guidelines and related standards. The use of registries can contribute to reducing the costs of providing care and improving the healthcare processes for patients and society [12]. To design the registry, it is essential to first extract the desired information items from different specialties and ensure agreement among various experts on these information items [13]. To ascertain accurate, comprehensive, and unambiguous information, it has been recommended to use minimum data sets [14], standard data collection tools, and guarantee access to precise and unambiguous health data of diseases [15]. Determining the minimum data set is used in various scientific fields; it is a standard and valid method for investigating diverse scientific subjects [16, 17, 18]. Therefore, this study aimed to determine the necessary minimum data set to establish a primary immunodeficiency disease registry.

## 2 Materials and Methods

This study was conducted in two stages, including a systematic review and a Quantitative Delphi Method. Ethical approval for this study was granted by the Ethics Committee of Shahid Beheshti University of Medical Sciences (IR.SBMU.RETECH.REC1402.018). The study adhered to all ethical guidelines and procedures for clinical research, ensuring the protection of participants' rights.

### 2.1 First Stage: Systematic Review

To determine the primary minimum data set, a systematic review method was conducted, following the Preferred Reporting Items for Systematic Reviews and Meta‐Analyses (PRISMA) guidelines [19]. The keywords “Primary Immunodeficiency”, “Diagnosis”, “Therapeutics” and “Disease Management” were used to search in PubMed, Web of Science, Scopus, and Cochrane databases. The search strategies in the mentioned databases are as follows:

*PubMed*

*(primary immunodeficiency[mesh]) AND (Diagnosis[mesh] OR Therapeutics[mesh] OR Disease Management[mesh])*

*Scopus*

((TITLE‐ABS‐KEY(“primary immunodeficiency”) OR TITLE‐ABS‐KEY(“congenital immunodeficiency”) OR TITLE‐ABS‐KEY(“inherited immunodeficiency”) OR TITLE‐ABS‐KEY(“primary immune deficiency”)) AND (TITLE‐ABS‐KEY(diagnosis) OR TITLE‐ABS‐KEY(diagnose*) OR TITLE‐ABS‐KEY(therapy) OR TITLE‐ABS‐KEY(therapies) OR TITLE‐ABS‐KEY(treatment*) OR TITLE‐ABS‐KEY(Therapeutics) OR TITLE‐ABS‐KEY(“Disease Management”)))

*Web of Science*

*TS* = ((“primary immunodeficiency” OR “congenital immunodeficiency” OR “inherited immunodeficiency” OR “primary immune deficiency”) AND (diagnosis OR diagnose* OR therapy OR therapies OR treatment* OR Therapeutics OR “Disease Management”))

*Cochrane*

“primary immunodeficiency” OR “congenital immunodeficiency” OR “inherited immunodeficiency” OR “primary immune deficiency”) AND (diagnosis OR diagnose* OR therapy OR therapies OR treatment* OR Therapeutics OR “Disease Management”)):ti, ab, kw

The records obtained from the aforementioned databases were imported into the EndNote software. In the first step, duplicate items were eliminated. Then, the remaining records were assessed based on their title and abstracts, and finally, their full texts were examined. The quality of the selected articles was evaluated by using the Strengthening the Reporting of Observational Studies in Epidemiology (STROBE) checklist, and articles which did not meet the required quality standards were excluded from the study [20]. The inclusion criteria included English documents that addressed data elements related to the diagnosis and/or treatment and/or management of primary immunodeficiency, and were available in full text. Exclusion criteria included non‐English articles, articles with limited accessibility, and articles not related to the diagnosis and/or treatment and/or management of primary immunodeficiency. After identifying the main documents, the relevant elements were extracted. Screening was conducted independently by two specialists. All disagreements were resolved through discussion. Finally, the extracted data elements were categorized into a questionnaire with two options: “necessary” and “not necessary” (See Supporting Information S1: Appendix 1). At the end of each section in the questionnaire, an open‐ended question was added to collect additional data elements.

### 2.2 Second Stage: Delphi Steps

This stage was conducted to validate the initial minimum data established in the previous step. It consisted of two rounds. The questionnaire designed in the previous stage was used for data collection. Then, items with scores above 75%, were included in the final minimum data set, and items with scores less than 50% were excluded. Items with scores between 50% and 75%, together with the suggested items in the first round, were used to develop a new questionnaire for the second round, which was then presented to the experts. Questions with scores above 75% in the second round were then added to the final minimum data set.

## 3 Results

### 3.1 First Stage: Systematic Review

**Figure 1:**
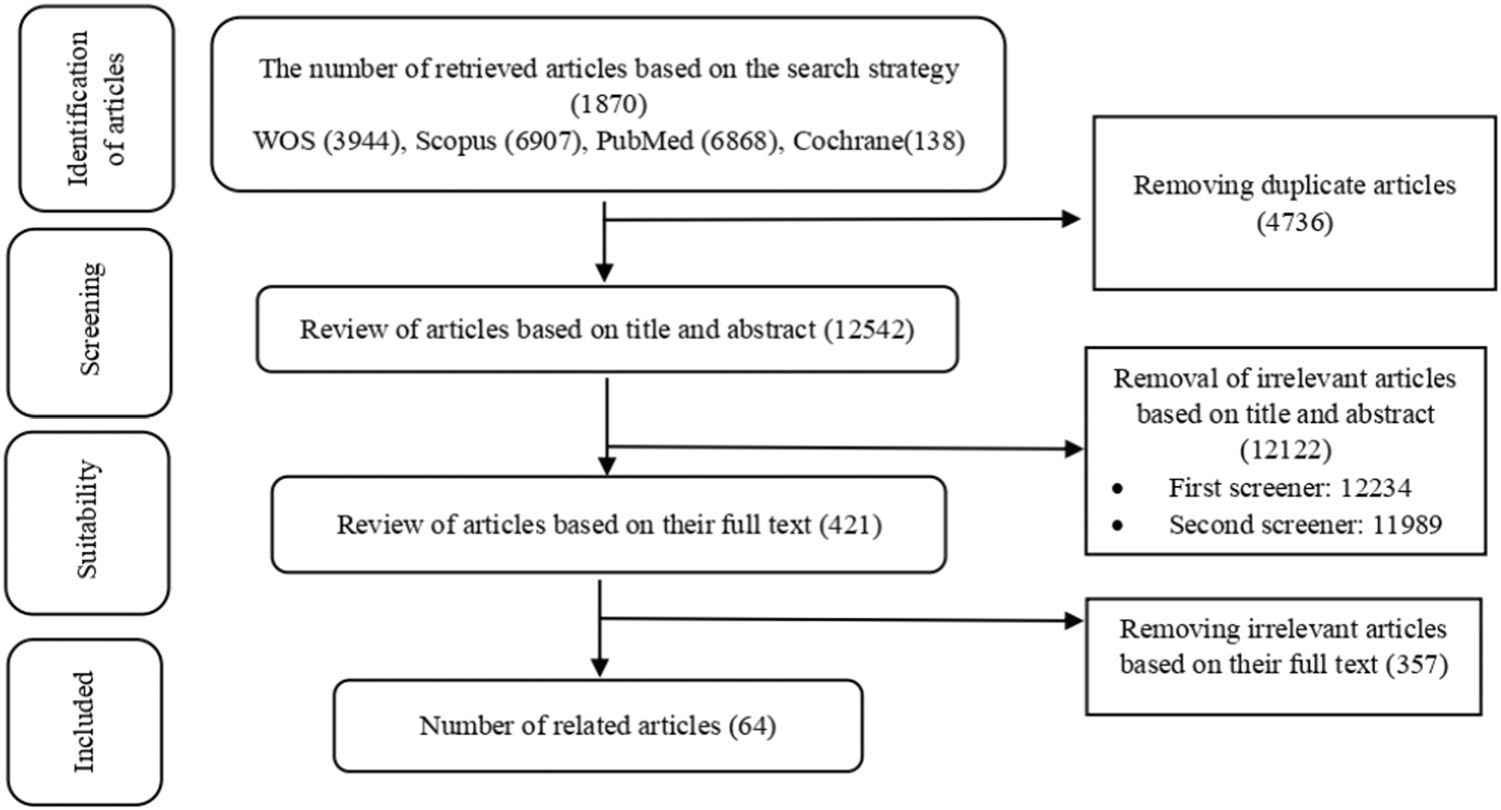
Study selection process.

The initial search in the mentioned databases yielded a total of 17,278 records. After eliminating 4736 duplicate records, the remaining 12,542 records underwent the review process. In the first stage, which focused on the title and abstract of the documents, 12,122 cases were excluded, leaving 421 cases for full text reading. At this stage, 357 documents were excluded based on the exclusion criteria, and 64 articles proceeded to the quality review stage. Finally, 56 articles met the required quality standards and were included in the study (See Supporting Information S1: Appendix 1). Figure 1 illustrates the review process.

In the next step, data elements related to the diagnosis, treatment, and management of primary immunodeficiency were extracted from the studies that met the inclusion criteria. Upon analysis, the extracted data elements were found to be divisible into two main sections: administrative and clinical. The administrative elements were further categorized into two sub‐sections: demographic and patient index. On the other hand, the clinical data were classified into four categories: patient history, physical examination, tests, and diagnosis (Table 1).

**Table 1:** Extracted data elements of the systematic review.

Sections	Sub‐sections			Data elements
Administration	Demographics			Name, surname, nationality, date of birth, gender, race, ethnicity, province of birth, telephone, address
	Patient index			National or passport number, medical record number, visit number
Clinical	History	Medical	Head and neck	Conjunctivitis, otitis media, otitis externa, pharyngitis, frequent colds, sinusitis, nasal polyps, oral thrush, oral candidiasis, thyroid disorder, neck lymph nodes
			Thoracic	Pneumonia, lung abscess, pleural effusion, pneumatocele, endocarditis, pericarditis
			Abdomen and pelvis	Frequent diarrhea, frequent constipation, frequent vomiting, blood in stool, dysphagia, odynophagia, epigastric pain, hepatomegaly, perianal disease, splenomegaly, visceral abscess
			Bone and soft tissue	Abscess cellulitis, osteomyelitis, chronic skin ulcer, fungal skin infection, herpes infection, muscle atrophy, late umbilical cord prolapse, omphalitis, eczema, petechiae, purpura, skin lesion
			Neurological and psychological	Meningitis, central nervous system abscess, encephalitis, seizures, ataxia, paresthesia, plegia, dysarthria, mental retardation, anxiety, panic, obsession, psychosis, depression
			Allergic	Food allergy, asthma, allergic rhinitis, atopic dermatitis, urticaria, drug allergy, IVIG allergy
			Others	Unusual bleeding, frequent bruising, autoimmune, malignancy, diabetes, hypertension, hyperlipidemia
		Vaccination		HPV, PPSV23, PCV13, VZV, rubella, measles, mumps, pertausis, tetanus, diphteria, HBV, OPV, BCG
		Medication		Prophylactic antibiotic, IVIG dose, IVIG/weight
		Family		Parental consanguinity, frequent infection in relatives (first and second degree), malignancy in relatives (first and second degree), autoimmunity in relatives (first and second degree), allergy in relatives (first and second degree), vaccine side effects in relatives (first and second degree)
		Social		Cigarette smoking, hookah smoking, opium smoking, alcohol consumption, drug consumption
	Physical examination	Vital signs		Blood pressure, pulse, body temperature, breathing
		Growth criteria		Weight, height, head circumference (before age 3)
		Organs health status		Tonsillomegaly, head and neck lymphadenopathy, thorax lymphadenopathy, abdominopelvic lymphadenopathy, inguinal lymphadenopathy, axillary lymphadenopathy, the shape of thorax, lung auscultation, heart auscultation, spleen size, liver size, eczema, warts, vitiligo, presence of fungal lesions, nail dysplasia, onychomycosis, clubbing
	Tests			CD3, CD4, CD8, CD16, CD56, CD19, CD20/22, CD45RO, CD11a, CD11b, CD11c, CD18, NBT, DHR, C3, C4, CH50, SGOT, SGPT, ALP, BUN, Cr, TSH, T4, FT4, sweat chloride test, genetic study for CF, Saccharin blue test, electron microscopy for immotile cilia, HIV1 Ab, HIV2 Ab, HIV RNA PCR, WBC, absolute PMN, absolute lymph, absolute Eo, absolute MONO, MPV, HCT, MCV MCH, Plt ESR, IgG, IgA, IgM, IgE, IgG1, IgG2, IgG3, IgG4, anti diphtheria Ab, anti tetanus Ab, anti pneumococcal Ab, mitogen in LTT, antigen in LTT, allogenic cell in LTT
	Diagnosis			Type of PID, age of PID onset, age of PID diagnosis, delay in PID diagnosis, diagnostic and therapeutic procedures, diagnostic and therapeutic surgeries, diagnostic and therapeutic medications

### 3.2 Second Stage: Two‐Round Delphi

The study included a sample of 10 asthma and allergy specialists from Hazrat Rasool Akram Hospital (See Supporting Information S1: Appendix 2), of whom 40% were female, while the remaining were male. The age range of the participants was between 40 and 62 years, with a mean age of 48.5 years, and their professional experience varied between 10 and 27 years, with an average of 15.4 years.

All participants in the study answered the questions. In the first round of the Delphi, eight data elements with scores between 50 and 75 were considered as essential and were included in the subsequent round of the Delphi. Additionally, a total of 13 data elements were suggested in the open question section at the conclusion of each round and were also included in the second round of the Delphi (Table 2). The Delphi results for each data element can be found in Supporting Information S1: Appendix 3.

**Table 2:** First round Delphi results.

First round Delphi results		Sections	Sub‐sections			Data elements
Agreed (score > 75)		Administration	Demographic			Name, surname, nationality, date of birth, gender, ethnicity, province of birth, province of birth, telephone, address
			Patient index			National or passport number, medical record number, visit number
		Clinical	History	Medical	Head and neck	Conjunctivitis, otitis media, otitis externa, pharyngitis, frequent colds, sinusitis, nasal polyps, oral thrush, oral candidiasis, thyroid disorder
					Thoracic	Pneumonia, lung abscess, pleural effusion, pneumatocele, endocarditis, pericarditis
					Abdomen and pelvis	Frequent diarrhea, frequent constipation, blood in stool, dysphagia, hepatomegaly, splenomegaly, visceral abscess
					Bone and soft tissue	Abscess cellulitis, osteomyelitis, fungal skin infection, herpes infection, late umbilical cord prolapse, omphalitis, eczema, purpura, skin lesion
					Neurological and psychological	Meningitis, central nervous system abscess, encephalitis, seizures, ataxia, paresthesia, plegia, dysarthria, mental retardation, panic, psychosis
					Allergic	Food allergy, asthma, allergic rhinitis, atopic dermatitis, urticaria, drug allergy, IVIG allergy
					Others	Unusual bleeding, frequent bruising, autoimmune, malignancy, diabetes, hypertension, hyperlipidemia
					Vaccination	HPV, PPSV23, PCV13, VZV, rubella, measles, mumps, pertausis, tetanus, diphteria, HBV, OPV, BCG
					Medication	Prophylactic antibiotic, IVIG dose, IVIG/weight
					Family	Parental consanguinity, frequent infection in relatives (first and second degree), malignancy in relatives (first and second degree), autoimmunity in relatives (first and second degree), allergy in relatives (first and second degree), vaccine side effects in relatives (first and second degree)
					Social	Cigarette smoking, hookah smoking, opium smoking, alcohol consumption, drug consumption
			Physical examination		Vital signs	Blood pressure, pulse, body temperature, breathing
					Growth criteria	Weight, height, head circumference (before age 3)
					Organs health status	Tonsillomegaly, head and neck lymphadenopathy, thorax lymphadenopathy, abdominopelvic lymphadenopathy, inguinal lymphadenopathy, axillary lymphadenopathy, the shape of thorax, lung auscultation, heart auscultation, spleen size, liver size, eczema, warts, vitiligo, presence of fungal lesions, nail dysplasia, onychomycosis, clubbing
					Tests	CD3, CD4, CD8, CD16, CD56, CD19, CD20/22, CD45RO, CD11a, CD11b, CD11c, CD18, NBT, DHR, C3, C4, CH50, SGOT, SGPT, ALP, BUN, Cr, TSH, T4, FT4, sweat chloride test, genetic study for CF, Saccharin blue test, electron microscopy for immotile cilia, HIV1 Ab, HIV2 Ab, HIV RNA PCR, WBC, absolute PMN, absolute lymph, absolute Eo, absolute MONO, MPV, HCT, MCV MCH, Plt ESR, IgG, IgA, IgM, IgE, IgG1, IgG2, IgG3, IgG4, Anti diphtheria Ab, anti tetanus Ab, anti pneumococcal Ab, mitogen in LTT, antigen in LTT, allogenic cell in LTT
					Diagnosis	Type of PID, age of PID onset, age of PID diagnosis, delay in PID diagnosis, diagnostic and therapeutic procedures, diagnostic and therapeutic surgeries, diagnostic and therapeutic medications
Second round	50 ≤ Score ≤ 75				Administrative	Race
		Clinical			Medical history	Neck lymph nodes, epigastric pain, perianal disease, chronic skin ulcer, anxiety, obsession, depression
	Proposed	Clinical			Medical history	Hepatomegaly, wart, rabies, flu
					Tests	CD45Ra, HGB, TCR x/b, TCR88, CD19, BCG, PHA, Candida
Removed (score < 50)					Administrative	province of birth, visit number
		Clinical			Medical history	Frequent vomiting, odynophagia, muscle atrophy, petechiae, purpura, pastysia

In the second round of the Delphi, one data element was excluded, while 20 data elements were included in the final minimum data sets (Table 3). The Delphi results for each data element are provided in Supporting Information S1: Appendix 4.

**Table 3:** Second round Delphi results.

Second round Delphi results	Data elements
Agreed (score ≥ 75)	Hepatomegaly, wart, rabies, flu, CD45Ra, HGB, TCR x/b, TCR88, CD19, BCG, PHA, candida, race, neck lymph nodes, perianal disease, chronic skin ulcer, anxiety, obsession, depression
Removed (score < 75)	Epigastric pain

## 4 Discussion

Creating a standardized registry to collect patient data is necessary to improve the quality and stability of clinical services, facilitate collaborative work, align treatment strategies, and create research opportunities [21]. So far, many registry systems for primary immunodeficiency have been established at the local and national levels. In Iran, the fourth report of the primary immune deficiency registry was published in 2018 [22]. However, no minimum data set has been reported for these registries. Given the inherent gap between data collection and their understanding and interpretation, it is essential to develop a minimum data set for primary immunodeficiency disease [16]. In this study, the primary immunodeficiency minimum data set was created through a systematic review and the Delphi method, which are standard and widely used methods to determine the minimum data sets. For example, Ahmadi et al. used this method to design the gestational diabetes minimal data set [7].

The minimum data set created in this study were categorized into two administrative (demographic and patient index) and clinical (patient history, physical examination, tests, and diagnosis) sections. Langarizadeh et al. also classified the minimum data set into two categories for rheumatoid arthritis and concluded that both administrative and clinical data are important for diagnosis and treatment [16].

González‐Costa et al.'s study indicated a relationship between gender and primary immunodeficiency disease, with men being 2.29 times more likely to be affected by the disease than women. Furthermore, the study showed a relationship between age and the onset of symptoms, with the average age of diagnosis being 11.89 years [23]. The current study identified age and gender as essential demographic factors.

Vincentiis et al.'s study highlighted ear, throat, and nose infections as important clinical manifestations of immunodeficiency. They found that 40% of individuals with immunodeficiency had a history of such infections, and that oral candidiasis and Hani's disease were the most common infection related to ear, nose and throat in children with immunodeficiency (60%–75%) followed by otitis media, sinusitis, and pharyngitis at a lower rate [24]. In this study, all manifestations (candidiasis, otitis media, sinusitis, and pharyngitis) were recognized as essential in the first round of the Delphi. This is consistent with the result of the study by Mazza and Lin on the relationship between sinusitis and immunodeficiency, where 50% of patients with chronic sinusitis suffered from immunodeficiency diseases [25].

In a study conducted by Jesenak et al., pulmonary manifestations such as pneumonia, rhinitis, bronchitis, and emphysema were more prominent in children with immunodeficiency disease [26]. Therefore, in the present study, the examination of these records is deemed necessary to facilitate diagnosis, treatment, and research of primary immunodeficiency disease.

Another study by Abdelhakim et al. on the skin manifestations of primary immunodeficiency disease highlighted bacterial and fungal skin infections as the most common skin manifestations associated with primary immunodeficiency, particularly in most primary immunodeficiency diseases caused by defects in T and B cells. Furthermore, eczema was observed in 19% of patients with primary immunodeficiency [27]. In the present study, the examination of the patient's history of bacterial and fungal infections is considered necessary for the diagnosis, treatment, and research of primary immunodeficiency disease.

Al‐Muhsen's systematic review on the gastrointestinal manifestations of primary immunodeficiency indicated various manifestations, such as diarrhea, vomiting, hepatomegaly, perianal problems, and splenomegaly in patients with this disease [4]. In the present study, the investigation of these gastrointestinal and liver problems is deemed necessary for the diagnosis, treatment, and research of primary immunodeficiency disease.

In a study on the neurological manifestations of primary immunodeficiency, Chavoshzadeh et al. observed manifestations, such as meningitis, ataxia, encephalitis, and psychosis in patients with immunodeficiency [28]. This study emphasizes the examination of these neurological problems for the diagnosis, treatment, and research of primary anemia.

Allergy is one of the first clinical manifestations in primary immunodeficiency. It may lead to delayed diagnosis or misdiagnosis in some cases [29]. Therefore, it is important to assess allergy history for accurate diagnosis and treatment of primary immunodeficiency disease.

Vaccination plays an important role in improving the quality of life, prognosis, and reducing infectious complications in patients with primary immunodeficiency. Khalili's review study confirms the effectiveness of all the vaccines considered essential in this study in treatment and treatment approach [30].

For over 20 years, IVIG has been used in the treatment of a wide range of primary and secondary immunodeficiencies. It is now a standard treatment option for most antibody deficiencies [31]. Thus, it is necessary to check the history of receiving IVIG.

A thorough examination of family medical history is crucial for diagnosing, treating, and reducing morbidity and mortality in patients with primary immunodeficiency disorders. In societies with a higher prevalence of parental consanguinity, the incidence of this disease is also increased [31]. Consequently, it is crucial to examine the parental consanguinity, autoimmune diseases, and immunodeficiency in first‐ and second‐degree relatives.

Smoking affects both innate and adaptive immunity and plays a dual role in immune regulation, through either exacerbating pathogenic immune responses or weakening defensive immunity. Generally, cigarette smoke weakens the body's immune system and makes it harder for the body to fight off infections. However, paradoxically, it can overstimulate the immune system and enhance autoimmunity [32]. Alcohol abuse can also result in medical complications, including changes in immune regulation that cause immunodeficiency and autoimmunity. Continuous alcohol consumption is associated with a decrease in the number of lymphocytes and an increased risk of bacterial and viral infections [33]. The current study also emphasizes the importance of considering social history, including the history of smoking, alcohol, and drug use, in the diagnosis and treatment of primary immunodeficiency disease.

The result of the study by Zhang et al. showed that patients with primary immunodeficiency had a significantly higher average body temperature in the morning, but there was no significant difference in their average body temperature in the evening or before bedtime [34]. A strong interaction between the immune and autonomic systems plays a prominent role in the initiation and maintenance of hypertension. Studies have shown consistent connections between high blood pressure, pro‐inflammatory cytokines, and immune system cells [35]. Primary immunodeficiency disease can affect the upper airways (such as sinusitis and otitis media) or the lower airways (such as pneumonia, bronchitis, bronchiectasis, and interstitial lung diseases), which can affect the respiratory rate [35]. This study also deems it necessary to examine vital signs for the diagnosis and treatment of primary immunodeficiency disease.

A higher percentage of patients with primary immunodeficiency exhibit physical growth disorders compared to healthy children. The study by Pieniawska‐Śmiech et al. revealed that patients with primary immunodeficiency had the lowest average weight and height at birth [36]. This study underscores the importance of examining growth indicators for the diagnosis and treatment of primary immunodeficiency disease.

Lymphadenopathy is one of the most important clinical symptoms of primary immunodeficiency. In a significant percentage of patients with primary immunodeficiency, lymph node involvement precedes other clinical features, leading to delays and complications in the diagnostic process [37]. Furthermore, primary immunodeficiency is associated with gastrointestinal manifestations such as diarrhea, vomiting, hepatomegaly, perianal problems, and splenomegaly [4]. This study also emphasizes the importance of conducting necessary examinations to evaluate lymphadenopathy in different organs as well as assessing the size of the spleen and liver, which is necessary for the diagnosis and treatment of primary immunodeficiency diseases.

Laboratory tests play a vital role in evaluating the function of the immune system of patients with immunodeficiency, specially when there is a history of frequent infections, unusual infections, or both. The appropriate and targeted use of immune function testing not only provides critical diagnostic information, but also helps decision‐making regarding the most appropriate treatment [38]. In their study “Laboratory evaluation of primary immunodeficiency”, Oliveira et al. concluded that immunoglobulin tests, complete blood count, flow cytometry tests, genetic tests, tests related to acquired immunodeficiency, routine tests, and antibody tests all have a diagnostic role (especially in case of differential diagnosis) [38]. Similarly, this study emphasizes these tests (a total of 57 tests) for the diagnosis and treatment of primary immunodeficiency disease.

Studies have demonstrated a delay of 8–24 months in the diagnosis of primary immunodeficiency. Additionally, it is estimated that approximately 70%–90% of individuals with existing primary immunodeficiency continue living without a definitive diagnosis [39]. This delay in diagnosis not only results in a delay in receiving specific treatments but also leads to increased complications, such as recurrent infections (e.g., pneumonia or sinusitis), poor quality of life, and even mortality [40]. Likewise, this study deems it necessary to examine the exact time of the diagnosis, delay in diagnosis, as well as the medical procedures taken to address it. Given the significance of these data from the perspective of experts and valid research, it is expected that this minimum data set will enhance decision‐making for physicians and policymakers and advance research in the field of primary immunodeficiency.

This study proposes a method for creating a registry based on expert consensus but does not provide evidence that this approach will be effective in practice. Additionally, a limitation of this approach is that it mines concepts from the literature, but some of the identified data elements may not be available in all health records, which could impact its practical implementation.

## Author Contributions

**Saman Mohammadpour:** conceptualization, investigation, funding acquisition, writing – original draft, writing – review and editing, visualization, methodology, validation, project administration, formal analysis, software, resources, supervision, data curation. **Hassan Emami:** conceptualization, investigation, methodology, validation, supervision, writing – original draft. **Sima Shokri:** conceptualization, methodology, resources, validation, supervision. **Rafat Bagherzadeh:** writing – original draft, writing – review and editing, methodology, validation. **Soqrat Omari Shekaftik:** writing – original draft, validation, methodology. **Zanko Hosseini:** writing – original draft, resources.

## Ethics Statement

This study was approved by the Ethics Committee of Shahid Beheshti University of Medical Sciences (IR.SBMU.RETECH.REC1402.018).

## Consent

Informed consent was obtained from all participants before data collection.

## Conflicts of Interest

The authors declare no conflicts of interest.

## Transparency Statement

The corresponding author, Hassan Emami, affirms that this manuscript is an honest, accurate, and transparent account of the study being reported; that no important aspects of the study have been omitted; and that any discrepancies from the study as planned (and, if relevant, registered) have been explained.

## Supporting information

Appendix.

## Data Availability

The authors confirm that the data supporting the findings of this study are available upon request from the corresponding author, Hassan Emami, via email.
