# Supplementary material for: Primary Immunodeficiency Registry System: The Minimum Data Set Designing Phase—A Systematic Review and Quantitative Delphi Study
Source: Health Sci Rep. 2025 Jul 9;8(7):e71015. doi: 10.1002/hsr2.71015 (PMC12241437; doi:10.1002/hsr2.71015)
Supplement: Supplementary file 1 — Appendix. [file HSR2-8-e71015-s001.docx]

Appendix 1: Final studies for the systematic review

| No | Author | Country/Year | Study type | Domain | Title | Ref |
| --- | --- | --- | --- | --- | --- | --- |
| 1 | Francisco A Bonilla | USA/2015 | Practice Guideline | diagnosis and management | Practice parameter for the diagnosis and management of primary immunodeficiency | (Bonilla et al., 2015) |
| 2 | Stephen Jolles | UK/2014 | Clinical Trial | treatment | Long-term efficacy, safety, and tolerability of Hizentra® for treatment of primary immunodeficiency disease | (Jolles et al., 2014) |
| 3 | Ebru Özdemir | Turkey/2023 | Cross-sectional | diagnosis | Retrospective evaluation of adults with primary immunodeficiency disease | (Özdemir, 2022) |
| 4 | Anoop Mayampurath | USA/2022 | cohort | diagnosis | Early Diagnosis of Primary Immunodeficiency Disease Using Clinical Data and Machine Learning | (Mayampurath et al., 2022) |
| 5 | [Andrew G Sikora](https://pubmed.ncbi.nlm.nih.gov/?size=200&term=Sikora+AG&cauthor_id=14567058) | USA/2003 | Review | diagnosis | Otolaryngologic manifestations of pediatric immunodeficiency | (Sikora and Lee, 2003) |
| 6 | Mazza JM | USA/2016 | Review | diagnosis | Primary immunodeficiency and recalcitrant chronic sinusitis: a systematic review | (Mazza and Lin, 2016) |
| 7 | Jesenak M | Slovakia/2014 | Review | diagnosis | Pulmonary manifestations of primary immunodeficiency disorders in children | (Jesenak et al., 2014) |
| 8 | L J Kobrynski | USA /2011 | Review | Diagnosis and treatment | Diagnosis and treatment of primary immunodeficiency disease in patients with gastrointestinal symptoms | (Kobrynski and Mayer, 2011) |
| 9 | Richard L Wasserman | USA/2011 | Review | Diagnosis and treatment | Diagnosis and treatment of primary immunodeficiency disease: the role of the otolaryngologist | (Wasserman and Manning, 2011) |
| 10 | Elif Soyak Aytekin | Turkey/2021 | Cross-sectional | diagnosis | Differential diagnosis of primary immunodeficiency in patients with BCGitis and BCGosis: A single-centre study | (Soyak Aytekin et al., 2021) |
| 11 | Hamoud Al-Mousa | Saudi Arabia/2017 | Review | Diagnosis and treatment  And management | Primary Immunodeficiency Diseases in Highly Consanguineous Populations from Middle East and North Africa: Epidemiology, Diagnosis, and Care | (Al-Mousa and Al-Saud, 2017) |
| 12 | Elena W Y Hsieh | USA/2016 | Review | Diagnosis | Novel tools for primary immunodeficiency diagnosis: making a case for deep profiling | (Hsieh and Hernandez, 2016) |
| 13 | Catherine Champi | USA/2002 | Review | Diagnosis and treatment | Primary immunodeficiency disorders in children: prompt diagnosis can lead to lifesaving treatment | (Champi, 2002) |
| 14 | Beatriz Costa-Carvalho | Mexico/2016 | Descriptive | diagnosis and treatment | Latin American challenges with the diagnosis and treatment of primary immunodeficiency diseases | (Costa-Carvalho et al., 2017) |
| 15 | Jennifer W Leiding | USA/2018 | Review | treatment | Precision medicine in the treatment of primary immunodeficiency diseases | (Leiding and Ballow, 2018) |
| 16 | Maurice R G O'gorman | USA/2007 | Review | diagnosis | Role of flow cytometry in the diagnosis and monitoring of primary immunodeficiency disease | (O'Gorman M, 2007) |
| 17 | [Hans D. Ochs](https://www.sciencedirect.com/author/7202761181/hans-dieter-ochs) | USA/2014 | review | diagnosis and treatment | Primary immunodeficiency disorders: general classification, new molecular insights, and practical approach to diagnosis and treatment | (Ochs and Hagin, 2014) |
| 18 | Won Kyung Kwon | Korea/2020 | Review | Diagnosis | Flow Cytometry for the Diagnosis of Primary Immunodeficiency Diseases: A Single Center Experience | (Kwon et al., 2020) |
| 19 | Vicki Modell | USA/2011 | Comparative Study | diagnosis and treatment | Global study of primary immunodeficiency diseases (PI)--diagnosis, treatment, and economic impact: an updated report from the Jeffrey Modell Foundation | (Modell et al., 2011) |
| 20 | [Wen-I Lee](https://pubmed.ncbi.nlm.nih.gov/?size=200&term=Lee+WI&cauthor_id=21782277) | Taiwan/2011 | Review | diagnosis and treatment | Distribution, clinical features and treatment in Taiwanese patients with symptomatic primary immunodeficiency diseases (PIDs) in a nationwide population-based study during 1985-2010 | (Lee et al., 2011) |
| 21 | [Anju Mishra](https://pubmed.ncbi.nlm.nih.gov/?size=200&term=Mishra+A&cauthor_id=24535004) | India/2014 | Cross-sectional | Diagnosis | Rapid Flow cytometric prenatal diagnosis of primary immunodeficiency (PID) disorders | (Mishra et al., 2014) |
| 22 | [Deirdre De Ranieri](https://pubmed.ncbi.nlm.nih.gov/?size=200&term=De+Ranieri+D&cauthor_id=28079912) | USA/2016 | Review | Treatment | Intravenous Immunoglobulin in the Treatment of Primary Immunodeficiency Diseases | (De Ranieri and Fenny, 2017) |
| 23 | [Rebecca H Buckle](https://pubmed.ncbi.nlm.nih.gov/?size=200&term=Buckley+RH&cauthor_id=16630930)y | USA/2006 | Case Reports | Diagnosis | Primary immunodeficiency or not? Making the correct diagnosis | (Buckley, 2006) |
| 24 | [Philip Wood](https://pubmed.ncbi.nlm.nih.gov/?size=200&term=Wood+P&cauthor_id=22547934) | United Kingdom/2012 | Review | Treatment | Human normal immunoglobulin in the treatment of primary immunodeficiency diseases | (Wood, 2012) |
| 25 | Francisco A Bonilla | USA/2005 | Guideline | diagnosis and treatment | Practice parameter for the diagnosis and management of primary immunodeficiency | (Bonilla et al., 2005) |
| 26 | Beatriz T Costa-Carvalho | Brazil/2018 | Cross-sectional | treatment | Low Rates of Poliovirus Antibodies in Primary Immunodeficiency Patients on Regular Intravenous Immunoglobulin Treatment | (Costa-Carvalho et al., 2018) |
| 27 | Luc Mouthon | French/2006 | Review | Diagnosis | Diagnosis of primary immunodeficiency in adult patients | (Mouthon et al., 2006) |
| 28 | Charlotte Cunningham-Rundle | USA/2004 | Cross-sectional | Diagnosis | Identifying undiagnosed primary immunodeficiency diseases in minority subjects by using computer sorting of diagnosis codes | (Cunningham-Rundles et al., 2004) |
| 29 | Fateme Sepehri | Iran/2017 | Letter to the Editor | Diagnosis | Developing Inference Model to Diagnosis of Primary Immunodeficiency Diseases in Protégé | (Sepehri et al., 2017) |
| 30 | U Nicolay | Sweden/2005 | Cross-sectional | treatment | Measuring treatment satisfaction in patients with primary immunodeficiency diseases receiving lifelong immunoglobulin replacement therapy | (Nicolay et al., 2005) |
| 31 | Shereen M Reda | UK/2015 | Cross-sectional | diagnosis | The importance of vaccination and immunoglobulin treatment for patients with primary immunodeficiency diseases (PIDs)--World PI Week April 22-29, 2015 | (Reda and Cant, 2015) |
| 32 | Pérsio Roxo-Junior | Brazil/2013 | Review | diagnosis | A family history of serious complications due to BCG vaccination is a tool for the early diagnosis of severe primary immunodeficiency | (Roxo-Junior et al., 2013) |
| 33 | Christopher C Lamb | USA/2019 | Cross sectional | decision | Shared decision making: Does a physician's decision-making style affect patient participation in treatment choices for primary immunodeficiency? | (Lamb et al., 2019) |
| 34 | Sweta S Shah | USA/2009 | Case Reports | Diagnosis | Diagnosis of primary immunodeficiency: let your eyes do the talking | (Shah et al., 2009) |
| 35 | Iris M Otani | USA/2022 | Guideline | diagnosis and management | Practical guidance for the diagnosis and management of secondary hypogammaglobulinemia: A Work Group Report of the AAAAI Primary Immunodeficiency and Altered Immune Response Committees | (Otani et al., 2022) |
| 36 | Jessica Quinn | USA/2022 | Case control | diagnosis and treatment | Growth in diagnosis and treatment of primary immunodeficiency within the global Jeffrey Modell Centers Network | (Quinn et al., 2022) |
| 37 | [Serdar Nepesov](https://pubmed.ncbi.nlm.nih.gov/?size=200&term=Nepesov+S&cauthor_id=35789402) | Turkey/2022 | Review | diagnosis | Diagnosis of primary immunodeficiency diseases in pediatric patients hospitalized for recurrent, severe, or unusual infections | (Nepesov et al., 2022) |
| 38 | Safa Abdelhakim | USA/2017 | Review | diagnosis | Cutaneous manifestations of primary immunodeficiency | (Abdelhakim et al., 2017) |
| 39 | Saleh Z. Al-Muhsen | Saudi Arabia/2010 | Review | diagnosis | Gastrointestinal and Hepatic Manifestations of Primary Immune Deficiency Diseases | (Al-Muhsen, 2010) |
| 40 | Zahra CHAVOSHZADEH | Iran/2018 | Review | diagnosis | Neurological Manifestations of Primary Immunodeficiencies | (Chavoshzadeh et al., 2018) |
| 41 | Zeinab A. El-Sayed | Egypt/2022 | Descriptive | diagnosis | Allergic manifestations of inborn errors of immunity and their impact on the diagnosis: A worldwide study | (El-Sayed et al., 2022) |
| 42 | Abbas Khalili | Iran/2020 | Review | Diagnosis and treatment | Vaccination in Patients with Primary Immunodeficiency Disorders | (Abolhassani et al., 2018) |
| 43 | SV Kaveri/2011 | France/2011 | Review | Diagnosis and treatment | Intravenous immunoglobulins in immunodeficiencies: more than mere replacement therapy | (Kaveri et al., 2011) |
| 44 | Mohamed A. Hendaus | Qatar/2014 | Case report | Diagnosis | The Value of Family History in Diagnosing Primary Immunodeficiency Disorders | (Hendaus et al., 2014) |
| 45 | Feifei Qiu | China/2017 | Review | diagnosis and management | Impacts of cigarette smoking on immune responsiveness: Up and down or upside down? | (Qiu et al., 2017) |
| 46 | Tasha Barr | USA/2015 | Review | diagnosis and management | Opposing Effects of Alcohol on the Immune System | (Barr et al., 2016) |
| 47 | Shouling Zhang | USA/2019 | cohort study | diagnosis and management | Body Temperature in Patients with Primary Immunodeficiency | (Zhang et al., 2019) |
| 48 | Madhu V Singh | USA/2014 | Review | diagnosis and management | The immune system and hypertension | (Singh et al., 2014) |
| 49 | Karolina Pieniawska-Śmiech | Poland/2020 | Cross sectional | diagnosis and management | Assessment of weight and height of patients with primary immunodeficiency disorders and group of children with recurrent respiratory tract infections | (Pieniawska-Śmiech et al., 2020) |
| 50 | Giorgio Costagliola | Italy/2021 | Review | diagnosis | Lymphadenopathy at the crossroad between immunodeficiency and autoinflammation: An intriguing challenge | (Costagliola and Consolini, 2021) |
| 51 | João B Oliveira | USA/2010 | Review | diagnosis | Laboratory evaluation of primary immunodeficiencies | (Oliveira and Fleisher, 2010) |
| 52 | Ahmad Bahrami | Iran/2020 | cross-sectional | diagnosis | Evaluation of the frequency and diagnostic delay of primary immunodeficiency disorders among suspected patients based on the 10 warning sign criteria: A cross-sectional study in Iran | (Bahrami et al., 2020) |
| 53 | John T Anderson | USA/2022 | Review | diagnosis and treatment | Health-related quality of life in primary immunodeficiencies: Impact of delayed diagnosis and treatment burden | (Anderson et al., 2022) |
| 54 | Lisa J Kobrynski | USA/2022 | Review | diagnosis | Newborn Screening in the Diagnosis of Primary Immunodeficiency | (Kobrynski, 2022) |
| 55 | Raha Zamani | Iran/2021 | Review | diagnosis | Primary immunodeficiency associated with hypopigmentation: A differential diagnosis approach | (Zamani et al., 2021) |
| 56 | K Toms | England/2021 | Cross sectional | diagnosis | Analysis of scoring systems for primary immunodeficiency diagnosis in adult immunology clinics | (Toms et al., 2021) |

Appendix 2: Details of Specialists Participating in the Delphi Process

| Specialist Name | Primary Specialty | Affiliation | Relevant Publications (if any) |
| --- | --- | --- | --- |
| Dr. Saba Arshi | Asthma & Allergy | Hazrat Rasool Akram Hospital | [Link](https://scholar.google.com/citations?user=ZgxmYV4AAAAJ&hl=fa) |
| Dr.Morteza Fallahpour | Allergist and Clinical immunologist | Hazrat Rasool Akram Hospital | [Link](https://scholar.google.com/citations?user=uzm8CkMAAAAJ&hl=en) |
| Dr.Sima Shokri | Allergy and Clinical Immunology | Hazrat Rasool Akram Hospital | [Link](https://scholar.google.com/citations?user=B9seJ3QAAAAJ&hl=en) |
| Dr. Mohammad Nabavi | Allergy and Clinical Immunology | Hazrat Rasool Akram Hospital | [Link](https://scholar.google.com/citations?user=UtsSVu0AAAAJ&hl=en) |
| Dr. Mohammad Hassan Bemanian | Allergy and Clinical Immunology | Hazrat Rasool Akram Hospital | [Link](https://scholar.google.com/citations?user=pVAqvlEAAAAJ&hl=en) |
| Dr. Sima Bahrami | Allergy and Clinical Immunology | Iran University of Medical Sciences | [Link](https://www.researchgate.net/profile/Sima-Bahrami) |
| Dr. Niusha Sharifinejad | Allergy and Clinical Immunology | Alborz University of Medical Sciences | [Link](https://scholar.google.com/citations?user=H5anW3oAAAAJ&hl=en) |
| Dr. Majid Khoshmirsafa | Allergy and Clinical Immunology | Iran University of Medical Sciences | [Link](https://scholar.google.com/citations?user=4R3kPTIAAAAJ&hl=en) |
| Dr. Farhad Seif | Allergy and Clinical Immunology | Alborz University of Medical Sciences | [Link](https://scholar.google.com/citations?hl=en&user=rFQN26gAAAAJ&view_op=list_works&sortby=pubdate) |
| Dr. Marzieh Tavakol | Allergy and Clinical Immunology | Alborz University of Medical Sciences | [Link](https://scholar.google.com/citations?user=l7-9xI4AAAAJ&hl=en) |

Appendix 3: Opinions of participants in the first round of Delphi for each data element

| Result | not necessary | necessary | Number of respondents | Data Element | Section | | | |
| --- | --- | --- | --- | --- | --- | --- | --- | --- |
|  | Number (percentage) | Number (percentage) |  |  |  |  |  |  |
| Agreed | 0(0) | 10(100) | 10 | Name | Demographic | | | Administration |
| Agreed | 0(0) | 10(100) | 10 | Surname |  |  |  |  |
| Agreed | 1(1) | 9(90) | 10 | Nationality |  |  |  |  |
| Agreed | 0(0) | 10(100) | 10 | Date of Birth |  |  |  |  |
| Agreed | 0(0) | 10(100) | 10 | Gender. |  |  |  |  |
| Second Round | 4(40) | 6(60) | 10 | Race. |  |  |  |  |
| Agreed | 2(20) | 8(80) | 10 | Ethnicity |  |  |  |  |
| Removed | 6(60) | 4(40) | 10 | Province of Birth |  |  |  |  |
| Agreed | 2(20) | 8(80) | 10 | Province of Birth |  |  |  |  |
| Agreed | 1(1) | 9(90) | 10 | Telephone |  |  |  |  |
| Agreed | 0(0) | 10(100) | 10 | Address |  |  |  |  |
| Agreed | 0(0) | 10(100) | 10 | National or passport number | Patient index | | |  |
| Agreed | 0(0) | 10(100) | 10 | Medical Record number |  |  |  |  |
| Removed | 6(60) | 4(40) | 10 | Visit number |  |  |  |  |
| Agreed | 0(0) | 10(100) | 10 | Conjunctivitis | Head and Neck | Medical | History | Clinical |
| Agreed | 0(0) | 10(100) | 10 | Otitis media |  |  |  |  |
| Agreed | 0(0) | 10(100) | 10 | Otitis externa |  |  |  |  |
| Agreed | 0(0) | 10(100) | 10 | Pharyngitis |  |  |  |  |
| Agreed | 2(20) | 8(80) | 10 | Frequent colds |  |  |  |  |
| Agreed | 0(0) | 10(100) | 10 | Sinusitis |  |  |  |  |
| Agreed | 1(1) | 9(90) | 10 | Nasal polyps |  |  |  |  |
| Agreed | 0(0) | 10(100) | 10 | Oral thrush |  |  |  |  |
| Agreed | 2(20) | 8(80) | 10 | Oral candidiasis |  |  |  |  |
| Second Round | 7(70) | 7(70) | 10 | Thyroid disorder |  |  |  |  |
| Agreed | 0(0) | 10(100) | 10 | neck lymph nodes |  |  |  |  |
| Agreed | 0(0) | 10(100) | 10 | Pneumonia | Thoracic |  |  |  |
| Agreed | 1(1) | 9(90) | 10 | Lung abscess |  |  |  |  |
| Agreed | 1(1) | 9(90) | 10 | Pleural effusion |  |  |  |  |
| Agreed | 0(0) | 10(100) | 10 | Pneumatocele |  |  |  |  |
| Agreed | 1(1) | 9(90) | 10 | Endocarditis |  |  |  |  |
| Agreed | 2(20) | 8(80) | 10 | Pericarditis |  |  |  |  |
| Agreed | 1(1) | 9(90) | 10 | Frequent diarrhea | Abdomen and Pelvis |  |  |  |
| Agreed | 2(20) | 8(80) | 10 | Frequent constipation |  |  |  |  |
| Second Round | 7(70) | 3(30) | 10 | frequent vomiting |  |  |  |  |
| Agreed | 2(20) | 8(80) | 10 | Blood in Stool |  |  |  |  |
| Agreed | 1(1) | 9(90) | 10 | Dysphagia |  |  |  |  |
| Removed | 6(60) | 4(40) | 10 | Odynophagia |  |  |  |  |
| Second Round | 4(40) | 6(60) | 10 | Epigastric pain |  |  |  |  |
| Agreed | 2(20) | 8(80) | 10 | Hepatomegaly |  |  |  |  |
| Second Round | 3(30) | 7(70) | 10 | Perianal Disease |  |  |  |  |
| Agreed | 2(20) | 8(80) | 10 | Splenomegaly |  |  |  |  |
| Agreed | 2(20) | 8(80) | 10 | Visceral abscess |  |  |  |  |
| Agreed | 0(0) | 10(100) | 10 | Abscess Cellulitis | Bone and Soft Tissue |  |  |  |
| Agreed | 1(1) | 9(90) | 10 | Osteomyelitis |  |  |  |  |
| Second Round | 3(30) | 7(70) | 10 | Chronic Skin Ulcer |  |  |  |  |
| Agreed | 2(20) | 8(80) | 10 | Fungal Skin Infection |  |  |  |  |
| Agreed | 2(20) | 8(80) | 10 | Ulcerate Skin Infection |  |  |  |  |
| Agreed | 1(1) | 9(90) | 10 | Herpes infection |  |  |  |  |
| Removed | 6(60) | 4(40) | 10 | Muscle atrophy |  |  |  |  |
| Agreed | 0(0) | 10(100) | 10 | Late Umbilical Cord Prolapse |  |  |  |  |
| Agreed | 0(0) | 10(100) | 10 | Omphalitis |  |  |  |  |
| Agreed | 1(1) | 9(90) | 10 | Eczema |  |  |  |  |
| Removed | 7(70) | 3(30) | 10 | Petechiae |  |  |  |  |
| Removed | 6(60) | 4(40) | 10 | Purpura |  |  |  |  |
| Agreed | 2(20) | 8(80) | 10 | Skin lesion |  |  |  |  |
| Agreed | 0(0) | 10(100) | 10 | Meningitis | Neurological and Psychological |  |  |  |
| Agreed | 0(0) | 10(100) | 10 | Central Nervous System Abscess |  |  |  |  |
| Agreed | 1(1) | 9(90) | 10 | Encephalitis |  |  |  |  |
| Agreed | 2(20) | 8(80) | 10 | Seizures |  |  |  |  |
| Agreed | 1(1) | 9(90) | 10 | Ataxia |  |  |  |  |
| Removed | 8(80) | 2(20) | 10 | Paresthesia |  |  |  |  |
| Agreed | 2(20) | 8(80) | 10 | Plegia |  |  |  |  |
| Agreed | 2(20) | 8(80) | 10 | Dysarthria |  |  |  |  |
| Agreed | 0(0) | 10(100) | 10 | Mental Retardation |  |  |  |  |
| Second Round | 3(30) | 7(70) | 10 | Anxiety |  |  |  |  |
| Agreed | 2(20) | 8(80) | 10 | Panic |  |  |  |  |
| Second Round | 3(30) | 7(70) | 10 | Obsession |  |  |  |  |
| Agreed | 2(20) | 8(80) | 10 | Psychosis |  |  |  |  |
| Second Round | 4(40) | 6(60) | 10 | Depression |  |  |  |  |
| Agreed | 0(0) | 10(100) | 10 | Food Allergy | Allergic |  |  |  |
| Agreed | 0(0) | 10(100) | 10 | Asthma |  |  |  |  |
| Agreed | 0(0) | 10(100) | 10 | Allergic Rhinitis |  |  |  |  |
| Agreed | 0(0) | 10(100) | 10 | Atopic Dermatitis |  |  |  |  |
| Agreed | 0(0) | 10(100) | 10 | Urticaria |  |  |  |  |
| Agreed | 0(0) | 10(100) | 10 | Drug Allergy, |  |  |  |  |
| Agreed | 0(0) | 10(100) | 10 | IVIG Allergy |  |  |  |  |
| Agreed | 0(0) | 10(100) | 10 | Unusual bleeding | Others |  |  |  |
| Agreed | 0(0) | 10(100) | 10 | Frequent Bruising |  |  |  |  |
| Agreed | 0(0) | 10(100) | 10 | Autoimmune |  |  |  |  |
| Agreed | 0(0) | 10(100) | 10 | Malignancy |  |  |  |  |
| Agreed | 1(1) | 9(90) | 10 | Diabetes |  |  |  |  |
| Agreed | 1(1) | 9(90) | 10 | Hypertension |  |  |  |  |
| Agreed | 0(0) | 10(100) | 10 | Hyperlipidemia |  |  |  |  |
| Agreed | 0(0) | 10(100) | 10 | HPV | Vaccination | |  |  |
| Agreed | 0(0) | 10(100) | 10 | PPSV23 |  |  |  |  |
| Agreed | 0(0) | 10(100) | 10 | PCV13 |  |  |  |  |
| Agreed | 0(0) | 10(100) | 10 | VZV |  |  |  |  |
| Agreed | 0(0) | 10(100) | 10 | rubell |  |  |  |  |
| Agreed | 0(0) | 10(100) | 10 | measles |  |  |  |  |
| Agreed | 0(0) | 10(100) | 10 | mumps. |  |  |  |  |
| Agreed | 0(0) | 10(100) | 10 | pertausis |  |  |  |  |
| Agreed | 0(0) | 10(100) | 10 | tetanus |  |  |  |  |
| Agreed | 0(0) | 10(100) | 10 | diphteria |  |  |  |  |
| Agreed | 0(0) | 10(100) | 10 | HBV |  |  |  |  |
| Agreed | 0(0) | 10(100) | 10 | OPV |  |  |  |  |
| Agreed | 0(0) | 10(100) | 10 | BCG |  |  |  |  |
| Agreed | 0(0) | 10(100) | 10 | Prophylactic antibiotic | Medication | |  |  |
| Agreed | 0(0) | 10(100) | 10 | IVIG dose |  |  |  |  |
| Agreed | 0(0) | 10(100) | 10 | IVIG/ weight |  |  |  |  |
| Agreed | 0(0) | 10(100) | 10 | Parental consanguinity | Family | |  |  |
| Agreed | 0(0) | 10(100) | 10 | Frequent Infection in relatives(first and second degree) |  |  |  |  |
| Agreed | 0(0) | 10(100) | 10 | Malignancy in relatives(first and second degree) |  |  |  |  |
| Agreed | 0(0) | 10(100) | 10 | Autoimmunity in relatives(first and second degree) |  |  |  |  |
| Agreed | 0(0) | 10(100) | 10 | Allergy in relatives(first and second degree) |  |  |  |  |
| Agreed | 0(0) | 10(100) | 10 | Vaccine side effects in relatives(first and second degree) |  |  |  |  |
| Agreed | 1(1) | 9(90) | 10 | Cigarette smoking | Social | |  |  |
| Agreed | 2(20) | 8(80) | 10 | Hookah smoking |  |  |  |  |
| Agreed | 1(1) | 9(90) | 10 | Opium smoking |  |  |  |  |
| Agreed | 1(1) | 9(90) | 10 | Alcohol consumption |  |  |  |  |
| Agreed | 2(20) | 8(80) | 10 | Drug consumption |  |  |  |  |
| Agreed | 0(0) | 10(100) | 10 | Blood Pressure | Vital Signs | | Physical Examination |  |
| Agreed | 0(0) | 10(100) | 10 | Pulse |  |  |  |  |
| Agreed | 0(0) | 10(100) | 10 | Body Temperature |  |  |  |  |
| Agreed | 0(0) | 10(100) | 10 | Breathing |  |  |  |  |
| Agreed | 0(0) | 10(100) | 10 | Weight | Growth criteria | |  |  |
| Agreed | 0(0) | 10(100) | 10 | Height |  |  |  |  |
| Agreed | 2(20) | 8(80) | 10 | Head Circumference(before age 3) |  |  |  |  |
| Agreed | 0(0) | 10(100) | 10 | Tonsillomegaly | Organs Health status | |  |  |
| Agreed | 0(0) | 10(100) | 10 | Head and Neck lymphadenopathy |  |  |  |  |
| Agreed | 0(0) | 10(100) | 10 | Thorax lymphadenopathy |  |  |  |  |
| Agreed | 0(0) | 10(100) | 10 | Abdominopelvic lymphadenopathy |  |  |  |  |
| Agreed | 0(0) | 10(100) | 10 | Inguinal lymphadenopathy |  |  |  |  |
| Agreed | 0(0) | 10(100) | 10 | Axillary lymphadenopathy |  |  |  |  |
| Agreed | 0(0) | 10(100) | 10 | The shape of thorax |  |  |  |  |
| Agreed | 0(0) | 10(100) | 10 | lung Auscultation |  |  |  |  |
| Agreed | 0(0) | 10(100) | 10 | Heart auscultation |  |  |  |  |
| Agreed | 0(0) | 10(100) | 10 | Spleen size |  |  |  |  |
| Agreed | 2(20) | 8(80) | 10 | Liver size |  |  |  |  |
| Agreed | 0(0) | 10(100) | 10 | Eczema |  |  |  |  |
| Agreed | 0(0) | 10(100) | 10 | warts |  |  |  |  |
| Agreed | 2(20) | 8(80) | 10 | vitiligo |  |  |  |  |
| Agreed | 0(0) | 10(100) | 10 | Presence of fungal lesions |  |  |  |  |
| Agreed | 1(1) | 9(90) | 10 | nail dysplasia |  |  |  |  |
| Agreed | 0(0) | 10(100) | 10 | onychomycosis |  |  |  |  |
| Agreed | 0(0) | 10(100) | 10 | clubbing |  |  |  |  |
| Agreed | 0(0) | 10(100) | 10 | CD3 | Tests | | |  |
| Agreed | 0(0) | 10(100) | 10 | CD4 |  |  |  |  |
| Agreed | 0(0) | 10(100) | 10 | CD8 |  |  |  |  |
| Agreed | 0(0) | 10(100) | 10 | CD16 |  |  |  |  |
| Agreed | 0(0) | 10(100) | 10 | CD56 |  |  |  |  |
| Agreed | 0(0) | 10(100) | 10 | CD19 |  |  |  |  |
| Agreed | 0(0) | 10(100) | 10 | CD20/22 |  |  |  |  |
| Agreed | 0(0) | 10(100) | 10 | CD45RO |  |  |  |  |
| Agreed | 0(0) | 10(100) | 10 | CD11a |  |  |  |  |
| Agreed | 0(0) | 10(100) | 10 | CD11b |  |  |  |  |
| Agreed | 0(0) | 10(100) | 10 | CD11c |  |  |  |  |
| Agreed | 0(0) | 10(100) | 10 | CD18 |  |  |  |  |
| Agreed | 2(20) | 8(80) | 10 | Mitogen in LTT |  |  |  |  |
| Agreed | 2(20) | 8(80) | 10 | Antigen in LTT |  |  |  |  |
| Agreed | 2(20) | 8(80) | 10 | Allogenic cell in LTT |  |  |  |  |
| Agreed | 2(20) | 8(80) | 10 | NBT |  |  |  |  |
| Agreed | 2(20) | 8(80) | 10 | DHR |  |  |  |  |
| Agreed | 2(20) | 8(80) | 10 | C3 |  |  |  |  |
| Agreed | 2(20) | 8(80) | 10 | C4 |  |  |  |  |
| Agreed | 2(20) | 8(80) | 10 | CH50 |  |  |  |  |
| Agreed | 2(20) | 8(80) | 10 | SGOT |  |  |  |  |
| Agreed | 2(20) | 8(80) | 10 | SGPT |  |  |  |  |
| Agreed | 2(20) | 8(80) | 10 | ALP |  |  |  |  |
| Agreed | 2(20) | 8(80) | 10 | BUN |  |  |  |  |
| Agreed | 2(20) | 8(80) | 10 | Cr |  |  |  |  |
| Agreed | 2(20) | 8(80) | 10 | TSH |  |  |  |  |
| Agreed | 2(20) | 8(80) | 10 | T4 |  |  |  |  |
| Agreed | 2(20) | 8(80) | 10 | FT4 |  |  |  |  |
| Agreed | 2(20) | 8(80) | 10 | Sweat chloride test |  |  |  |  |
| Agreed | 2(20) | 8(80) | 10 | Genetic study for CF |  |  |  |  |
| Agreed | 2(20) | 8(80) | 10 | Saccharin blue test |  |  |  |  |
| Agreed | 2(20) | 8(80) | 10 | Electron microscopy for immotile cilia |  |  |  |  |
| Agreed | 0(0) | 10(100) | 10 | HIV1 Ab |  |  |  |  |
| Agreed | 0(0) | 10(100) | 10 | HIV2 Ab |  |  |  |  |
| Agreed | 0(0) | 10(100) | 10 | HIV RNA PCR |  |  |  |  |
| Agreed | 0(0) | 10(100) | 10 | WBC |  |  |  |  |
| Agreed | 0(0) | 10(100) | 10 | Absolute PMN |  |  |  |  |
| Agreed | 0(0) | 10(100) | 10 | Absolute Lymph |  |  |  |  |
| Agreed | 0(0) | 10(100) | 10 | Absolute Eo |  |  |  |  |
| Agreed | 0(0) | 10(100) | 10 | Absolute MONO |  |  |  |  |
| Agreed | 0(0) | 10(100) | 10 | MPV |  |  |  |  |
| Agreed | 0(0) | 10(100) | 10 | HCT |  |  |  |  |
| Agreed | 0(0) | 10(100) | 10 | MCV |  |  |  |  |
| Agreed | 0(0) | 10(100) | 10 | MCH |  |  |  |  |
| Agreed | 0(0) | 10(100) | 10 | Plt |  |  |  |  |
| Agreed | 1(1) | 9(90) | 10 | ESR |  |  |  |  |
| Agreed | 0(0) | 10(100) | 10 | IgG |  |  |  |  |
| Agreed | 0(0) | 10(100) | 10 | IgA |  |  |  |  |
| Agreed | 0(0) | 10(100) | 10 | IgM |  |  |  |  |
| Agreed | 0(0) | 10(100) | 10 | IgE |  |  |  |  |
| Agreed | 0(0) | 10(100) | 10 | IgG1 |  |  |  |  |
| Agreed | 0(0) | 10(100) | 10 | IgG2 |  |  |  |  |
| Agreed | 0(0) | 10(100) | 10 | IgG3 |  |  |  |  |
| Agreed | 0(0) | 10(100) | 10 | IgG4 |  |  |  |  |
| Agreed | 0(0) | 10(100) | 10 | Anti diphtheria Ab |  |  |  |  |
| Agreed | 0(0) | 10(100) | 10 | Anti tetanus Ab |  |  |  |  |
| Agreed | 0(0) | 10(100) | 10 | Anti pneumococcal Ab |  |  |  |  |
| Agreed | 0(0) | 10(100) | 10 | Type of PID | Diagnosis | | |  |
| Agreed | 0(0) | 10(100) | 10 | Age of PID onset |  |  |  |  |
| Agreed | 0(0) | 10(100) | 10 | Age of PID diagnosis |  |  |  |  |
| Agreed | 0(0) | 10(100) | 10 | Delay in PID diagnosis |  |  |  |  |
| Agreed | 0(0) | 10(100) | 10 | Diagnostic and therapeutic procedures |  |  |  |  |
| Agreed | 0(0) | 10(100) | 10 | Diagnostic and therapeutic surgeries |  |  |  |  |
| Agreed | 1(1) | 9(90) | 10 | Diagnostic and therapeutic medications |  |  |  |  |

Appendix 4: Opinions of participants in Second Round of Delphi for each data element

| Result | Not-necessary | necessary | Number of respondents | Data Element | Section | |
| --- | --- | --- | --- | --- | --- | --- |
|  | Number (percentage) | Number (percentage) |  |  |  |  |
| Agreed | 2(20) | 8(80) | 10 | Race | Demographics | Administrative |
| Agreed | 1(10) | 9(90) | 10 | Neck lymph nodes | History | Clinical |
| Removed | 5(50) | 5(50) | 10 | Epigastric pain |  |  |
| Agreed | 2(20) | 8(80) | 10 | Perianal problems |  |  |
| Agreed | 1(10) | 9(90) | 10 | Chronic skin ulcer |  |  |
| Agreed | 2(20) | 8(80) | 10 | Anxiety |  |  |
| Agreed | 2(20) | 8(80) | 10 | Obsession |  |  |
| Agreed | 2(20) | 8(80) | 10 | Depression |  |  |
| Agreed | 2(20) | 8(80) | 10 | Hepatomegaly |  |  |
| Agreed | 1(10) | 9(90) | 10 | wart |  |  |
|  | 1(10) | 9(90) | 10 | IPV |  |  |
| Agreed | 0(0) | 10(100) | 10 | Rabies |  |  |
| Agreed | 1(10) | 9(90) | 10 | Flu |  |  |
| Agreed | 0(0) | 10(100) | 10 | CD45Ra | Tests |  |
| Agreed | 0(0) | 10(100) | 10 | HGB |  |  |
| Agreed | 0(0) | 10(100) | 10 | TCRx/b |  |  |
| Agreed | 0(0) | 10(100) | 10 | TCR88 |  |  |
| Agreed | 0(0) | 10(100) | 10 | CD19 |  |  |
| Agreed | 0(0) | 10(100) | 10 | BCG |  |  |
| Agreed | 0(0) | 10(100) | 10 | PHA |  |  |
| Agreed | 1(10) | 9(90) | 10 | Candida |  |  |

Abdelhakim S, Cafone J and Basak RB (2017) Cutaneous manifestations of primary immunodeficiency. *Indian Journal of Paediatric Dermatology* 18(3): 155-159.

Abolhassani H, Kiaee F, Tavakol M, et al. (2018) Fourth Update on the Iranian National Registry of Primary Immunodeficiencies: Integration of Molecular Diagnosis. *J Clin Immunol* 38(7): 816-832.

Al-Mousa H and Al-Saud B (2017) Primary Immunodeficiency Diseases in Highly Consanguineous Populations from Middle East and North Africa: Epidemiology, Diagnosis, and Care. *Front Immunol* 8: 678.

Al-Muhsen SZ (2010) Gastrointestinal and hepatic manifestations of primary immune deficiency diseases. *Saudi J Gastroenterol* 16(2): 66-74.

Anderson JT, Cowan J, Condino-Neto A, et al. (2022) Health-related quality of life in primary immunodeficiencies: Impact of delayed diagnosis and treatment burden. *Clinical Immunology* 236: 108931.

Bahrami A, Sayyahfar S, Soltani Z, et al. (2020) Evaluation of the frequency and diagnostic delay of primary immunodeficiency disorders among suspected patients based on the 10 warning sign criteria: A cross-sectional study in Iran. *ALLERGOLOGIA ET IMMUNOPATHOLOGIA* 48(6): 711-719.

Barr T, Helms C, Grant K, et al. (2016) Opposing effects of alcohol on the immune system. *Progress in Neuro-Psychopharmacology and Biological Psychiatry* 65: 242-251.

Bonilla FA, Bernstein IL, Khan DA, et al. (2005) Practice parameter for the diagnosis and management of primary immunodeficiency. *Ann Allergy Asthma Immunol* 94(5 Suppl 1): S1-63.

Bonilla FA, Khan DA, Ballas ZK, et al. (2015) Practice parameter for the diagnosis and management of primary immunodeficiency. *J Allergy Clin Immunol* 136(5): 1186-1205.e1181-1178.

Buckley RH (2006) Primary immunodeficiency or not? Making the correct diagnosis. *J Allergy Clin Immunol* 117(4): 756-758.

Champi C (2002) Primary immunodeficiency disorders in children: prompt diagnosis can lead to lifesaving treatment. *J Pediatr Health Care* 16(1): 16-21.

Chavoshzadeh Z, Hashemitari A and Darougar S (2018) Neurological Manifestations of Primary Immunodeficiencies. *Iran J Child Neurol* 12(3): 7-23.

Costa-Carvalho B, González-Serrano M, Espinosa-Padilla S, et al. (2017) Latin American challenges with the diagnosis and treatment of primary immunodeficiency diseases. *Expert Rev Clin Immunol* 13(5): 483-489.

Costa-Carvalho BT, Sullivan KE, Fontes PM, et al. (2018) Low Rates of Poliovirus Antibodies in Primary Immunodeficiency Patients on Regular Intravenous Immunoglobulin Treatment. *J Clin Immunol* 38(5): 628-634.

Costagliola G and Consolini R (2021) Lymphadenopathy at the crossroad between immunodeficiency and autoinflammation: An intriguing challenge. *Clin Exp Immunol* 205(3): 288-305.

Cunningham-Rundles C, Sidi P, Estrella L, et al. (2004) Identifying undiagnosed primary immunodeficiency diseases in minority subjects by using computer sorting of diagnosis codes. *J Allergy Clin Immunol* 113(4): 747-755.

De Ranieri D and Fenny NS (2017) Intravenous Immunoglobulin in the Treatment of Primary Immunodeficiency Diseases. *Pediatr Ann* 46(1): e8-e12.

El-Sayed ZA, El-Ghoneimy DH, Ortega-Martell JA, et al. (2022) Allergic manifestations of inborn errors of immunity and their impact on the diagnosis: A worldwide study. *World Allergy Organ J* 15(6): 100657.

Hendaus MA, Alhammadi A, Adeli MM, et al. (2014) The value of family history in diagnosing primary immunodeficiency disorders. *Case Rep Pediatr* 2014: 516256.

Hsieh EW and Hernandez JD (2016) Novel tools for primary immunodeficiency diagnosis: making a case for deep profiling. *Curr Opin Allergy Clin Immunol* 16(6): 549-556.

Jesenak M, Banovcin P, Jesenakova B, et al. (2014) Pulmonary manifestations of primary immunodeficiency disorders in children. *Front Pediatr* 2: 77.

Jolles S, Borte M, Nelson RP, Jr., et al. (2014) Long-term efficacy, safety, and tolerability of Hizentra® for treatment of primary immunodeficiency disease. *Clin Immunol* 150(2): 161-169.

Kaveri SV, Maddur MS, Hegde P, et al. (2011) Intravenous immunoglobulins in immunodeficiencies: more than mere replacement therapy. *Clin Exp Immunol* 164 Suppl 2(Suppl 2): 2-5.

Kobrynski LJ (2022) Newborn Screening in the Diagnosis of Primary Immunodeficiency. *CLINICAL REVIEWS IN ALLERGY & IMMUNOLOGY* 63(1): 9-21.

Kobrynski LJ and Mayer L (2011) Diagnosis and treatment of primary immunodeficiency disease in patients with gastrointestinal symptoms. *Clin Immunol* 139(3): 238-248.

Kwon WK, Choi S, Kim HJ, et al. (2020) Flow Cytometry for the Diagnosis of Primary Immunodeficiency Diseases: A Single Center Experience. *Allergy Asthma Immunol Res* 12(2): 292-305.

Lamb CC, Wang Y and Lyytinen K (2019) Shared decision making: Does a physician's decision-making style affect patient participation in treatment choices for primary immunodeficiency? *J Eval Clin Pract* 25(6): 1102-1110.

Lee WI, Huang JL, Jaing TH, et al. (2011) Distribution, clinical features and treatment in Taiwanese patients with symptomatic primary immunodeficiency diseases (PIDs) in a nationwide population-based study during 1985-2010. *Immunobiology* 216(12): 1286-1294.

Leiding JW and Ballow M (2018) Precision medicine in the treatment of primary immunodeficiency diseases. *Curr Opin Allergy Clin Immunol* 18(2): 159-166.

Mayampurath A, Ajith A, Anderson-Smits C, et al. (2022) Early Diagnosis of Primary Immunodeficiency Disease Using Clinical Data and Machine Learning. *J Allergy Clin Immunol Pract* 10(11): 3002-3007.e3005.

Mazza JM and Lin SY (2016) Primary immunodeficiency and recalcitrant chronic sinusitis: a systematic review. *Int Forum Allergy Rhinol* 6(10): 1029-1033.

Mishra A, Gupta M, Dalvi A, et al. (2014) Rapid Flow cytometric prenatal diagnosis of primary immunodeficiency (PID) disorders. *J Clin Immunol* 34(3): 316-322.

Modell V, Gee B, Lewis DB, et al. (2011) Global study of primary immunodeficiency diseases (PI)--diagnosis, treatment, and economic impact: an updated report from the Jeffrey Modell Foundation. *Immunol Res* 51(1): 61-70.

Mouthon L, Berezné A, Guillevin L, et al. (2006) [Diagnosis of primary immunodeficiency in adult patients]. *Presse Med* 35(5 Pt 2): 903-911.

Nepesov S, Firtina S, Aygun FD, et al. (2022) Diagnosis of primary immunodeficiency diseases in pediatric patients hospitalized for recurrent, severe, or unusual infections. *Allergol Immunopathol (Madr)* 50(4): 50-56.

Nicolay U, Haag S, Eichmann F, et al. (2005) Measuring treatment satisfaction in patients with primary immunodeficiency diseases receiving lifelong immunoglobulin replacement therapy. *Qual Life Res* 14(7): 1683-1691.

O'Gorman M R (2007) Role of flow cytometry in the diagnosis and monitoring of primary immunodeficiency disease. *Clin Lab Med* 27(3): 591-626, vii.

Ochs HD and Hagin D (2014) Primary immunodeficiency disorders: general classification, new molecular insights, and practical approach to diagnosis and treatment. *Ann Allergy Asthma Immunol* 112(6): 489-495.

Oliveira JB and Fleisher TA (2010) Laboratory evaluation of primary immunodeficiencies. *J Allergy Clin Immunol* 125(2 Suppl 2): S297-305.

Otani IM, Lehman HK, Jongco AM, et al. (2022) Practical guidance for the diagnosis and management of secondary hypogammaglobulinemia: A Work Group Report of the AAAAI Primary Immunodeficiency and Altered Immune Response Committees. *J Allergy Clin Immunol* 149(5): 1525-1560.

Özdemir E (2022) Retrospective evaluation of adults with primary immunodeficiency disease. *Postepy Dermatol Alergol* 39(5): 976-979.

Pieniawska-Śmiech K, Bar K, Babicki M, et al. (2020) Assessment of weight and height of patients with primary immunodeficiency disorders and group of children with recurrent respiratory tract infections. *BMC Immunol* 21(1): 42.

Qiu F, Liang CL, Liu H, et al. (2017) Impacts of cigarette smoking on immune responsiveness: Up and down or upside down? *Oncotarget* 8(1): 268-284.

Quinn J, Modell V, Orange JS, et al. (2022) Growth in diagnosis and treatment of primary immunodeficiency within the global Jeffrey Modell Centers Network. *Allergy Asthma Clin Immunol* 18(1): 19.

Reda SM and Cant AJ (2015) The importance of vaccination and immunoglobulin treatment for patients with primary immunodeficiency diseases (PIDs)--World PI Week April 22-29, 2015. *Eur J Immunol* 45(5): 1285-1286.

Roxo-Junior P, Silva J, Andrea M, et al. (2013) A family history of serious complications due to BCG vaccination is a tool for the early diagnosis of severe primary immunodeficiency. *Ital J Pediatr* 39: 54.

Sepehri F, Langarizadeh M, Sharifi L, et al. (2017) Developing Inference Model to Diagnosis of Primary Immunodeficiency Diseases in Protégé. *Acta Med Iran* 55(4): 280-281.

Shah SS, Bacino CA, Sheehan AM, et al. (2009) Diagnosis of primary immunodeficiency: let your eyes do the talking. *J Allergy Clin Immunol* 124(6): 1363-1364.e1361.

Sikora AG and Lee KC (2003) Otolaryngologic manifestations of immunodeficiency. *Otolaryngol Clin North Am* 36(4): 647-672.

Singh MV, Chapleau MW, Harwani SC, et al. (2014) The immune system and hypertension. *Immunol Res* 59(1-3): 243-253.

Soyak Aytekin E, Keskin A, Tan C, et al. (2021) Differential diagnosis of primary immunodeficiency in patients with BCGitis and BCGosis: A single-centre study. *Scand J Immunol* 94(4): e13084.

Toms K, Gkrania-Klotsas E and Kumararatne D (2021) Analysis of scoring systems for primary immunodeficiency diagnosis in adult immunology clinics. *CLINICAL AND EXPERIMENTAL IMMUNOLOGY* 203(1): 47-54.

Wasserman RL and Manning SC (2011) Diagnosis and treatment of primary immunodeficiency disease: the role of the otolaryngologist. *Am J Otolaryngol* 32(4): 329-337.

Wood P (2012) Human normal immunoglobulin in the treatment of primary immunodeficiency diseases. *Ther Clin Risk Manag* 8: 157-167.

Zamani R, Shahkarami S and Rezaei N (2021) Primary immunodeficiency associated with hypopigmentation: A differential diagnosis approach. *ALLERGOLOGIA ET IMMUNOPATHOLOGIA* 49(2): 178-190.

Zhang S, Henderson TS, Scalchunes C, et al. (2019) Body Temperature in Patients with Primary Immunodeficiency. *JOURNAL OF CLINICAL IMMUNOLOGY.* SPRINGER/PLENUM PUBLISHERS 233 SPRING ST, NEW YORK, NY 10013 USA, S35-S35.
